# Ultra-stable Mn_1-__x_Ni_x_CO_3_ nano/sub-microspheres positive electrodes for high-performance solid-state asymmetric supercapacitors

**DOI:** 10.1038/s41598-020-64867-8

**Published:** 2020-06-01

**Authors:** Srinivasan Alagar, Rajesh Madhuvilakku, Ramalakshmi Mariappan, Chelladurai Karuppiah, Chun-Chen Yang, Shakkthivel Piraman

**Affiliations:** 1 Sustainable Energy and Smart Materials Research Lab, Department of Nanoscience and Technology, Science Campus, Alagappa University, Karaikudi, 630002 Tamil Nadu India; 2 Battery Research Center of Green Energy, Ming Chi University of Technology, New Taipei City, 24301 ROC Taiwan

**Keywords:** Supercapacitors, Materials science, Nanoscience and technology

## Abstract

Long–term cycling performance of electrodes for application in supercapcitor has received large research interest in recent years. Ultra-stable Mn_1-__x_Ni_x_CO_3_ (x-0, 0.20, 0.25 and 0.30) nano/sub-microspheres were synthesized via simple co-precipitation method and the Mn_1-_xNi_x_CO_3_ was confirmed by XRD, FT-IR, XPS and their morphology was studied by SEM and TEM analysis. Among the various Mn_1-x_Ni_x_CO_3_ electrodes, the Mn_0.75_Ni_0.25_CO_3_ electrode exhibited the higher specific capacitance (364 F g^−1^ at 1 A g^−1^) with capacity retention of 96% after 7500 cycles at 5 A g^−1^. Moreover, the assembled solid-state asymmetric supercapacitor based on Mn_0.75_Ni_0.25_CO_3_//graphene nanosheets performed a high specific capacity of 46 F g^−1^ and energy density of 25 Wh kg^−1^ at a power density of 499 W kg^−1^ along with high capacity retention of 87.7% after 7500 cycles. The improved electrochemical performances are mainly owing to the intrinsic conductivity and electrochemical activity of MnCO_3_ after Mn_1-__x_Ni_x_CO_3_ (x-0.20, 0.25 and 0.30) with appropriate Ni concentration. This study highlights the potentiality of the Mn_0.75_Ni_0.25_CO_3_//GNS asymmetric supercapacitor device for promising energy storage applications.

## Introduction

In the 21^st^ century, there is an urge to develop novel new energy storage technology to meet the demand of highly sustained energy storage systems. Interest on the development of low cost, high energy density and power density electrochemical energy storage systems compatible with nuclear, solar and wind energy resources are growing tremendously particularly on supercapacitors and batteries^1–3^, former one satisfy all the requirements. Whereas, conventional capacitors deliver high power, but lower energy density which instigates the search for long-term cyclic stability, operating safety, environmental friendly, low cost and can deliver high power and energy density supercapacitors^4–7^. The prominent features of supercapacitors are long-term cyclic stability, operating safety, environment friendly, and can deliver high power density. The supercapacitors can be classified in to two types such as; (i) EDLC (electrical double layer capacitors) and (ii) pseudocapacitors which is differentiated by their charge-storage mechanism^8,9^. In EDLC charge is stored by rapid adsorption/ desorption at the electrode/electrolyte interface, there is no redox reaction occurs in the electrode materials but the specific capacitance is very lower which depends on the electrode active surface area, whereas the surface of the electrode materials will undergo a reversible redox (Faradaic) reaction in the pseudocapacitors during the charge – discharge processes particularly in the metal oxide or conducting polymer electrode materials. EDLC offers lower capacitance which necessitate to identify a suitable pseudocapacitor materials (transition metal oxides) that can able to store more charges for longer period without noticeable energy loss^10,11^.

The transition metal oxides have received great attention towards pseudocapacitor materials owing to their variable oxidation states and ease of preparation. Among the various metal oxides, RuO_2. X_H_2_O is a well-known pseudocapacitor with good performance and have high specific capacitance (760 F g^−1^) with enhanced cycle-life. However, the low porosity, toxicity, less abundance of its raw materials and rapid decrease in power density at high charge- discharge current limit, restrict its glassine applications^12,13^. A large number of electrochemical studies are focused in finding a low-cost metal oxide as a replacement for RuO_2_. Recently, enormous effort has been focused on transition metal compounds containing nickel, cobalt, manganese with different morphologies. Wang and his co-workers synthesized interlinked multiphase Fe-doped MnO_2_ nanostructures with electrochemical properties^14^. Chen *et al*. have synthesized NiCo_2_O_4_@NiWO_3_ nanowire arrays that can serves as an electroactive material for super capacitors and it delivers a high capacity retention and long-term cycle stability^15^. Zhang *et al*. fabricated 3D Co_3_O_4_–Ni_3_(VO_4_)_2_ heterostructured nanorods on nickel foam possessing improved electrochemical properties for supercapacitor electrodes and exhibits good comprehensive electrochemical performance^16^. These transitional electrode materials with high specific surface area have been widely used as pseudocapacitor applications^17–19^. In general, aforementioned reports illustrate the use of transition metal oxides, sulphides and hydroxides as the supercapacitors electrode materials, only a few electrodes with metal carbonates are reported^20–26^. However, to the best of our knowledge, first time we have reported the synthesis of Mn_0.75_Ni_0.25_CO_3_ nano/sub-microspheres and applied as electrode materials for solid-state asymmetric supercapacitor (ASC) applications, since it is environment friendly, and can be easily fabricated with capacitance performance.

In this work, we have adopted a facile template-free co-precipitation method assisted with sodium bicarbonate to synthesize the shape-controlled Mn_1-__x_Ni_x_CO_3_ (x-0, 0.20, 0.25 and 0.30) nano/sub-microspheres. The crystalline structure, functional groups and morphology of the as-prepared materials were characterized by X-ray diffraction (XRD), Fourier transform infrared spectroscopy (FT-IR), scanning electron microscopy (SEM) and transmission electron microscopy (TEM) studies. The electrochemical studies show that the Mn_0.75_Ni_0.25_CO_3_ nano/sub-microspheres exhibits a higher specific capacitance of 364 F g^−1^ at a current density of 1 A g^−1^, high rate capability and superior cyclic stability. In addition, the solid-state ASC with Mn_0.75_Ni_0.25_CO_3_//GNS configuration was fabricated device using Mn_0.75_Ni_0.25_CO_3_ nano/sub-microspheres and graphene nanosheets (GNS) as positive and negative electrodes, respectively. The solid-state ASC Mn_0.75_Ni_0.25_CO_3_//GNS device exhibits a high energy density of 25 Wh kg^−1^ with a power density of 499 W kg^−1^. The fabricated Mn_0.75_Ni_0.25_CO_3_//GNS configuration is a potential system for commercial applications.

## Results and discussion

**Figure 1:**
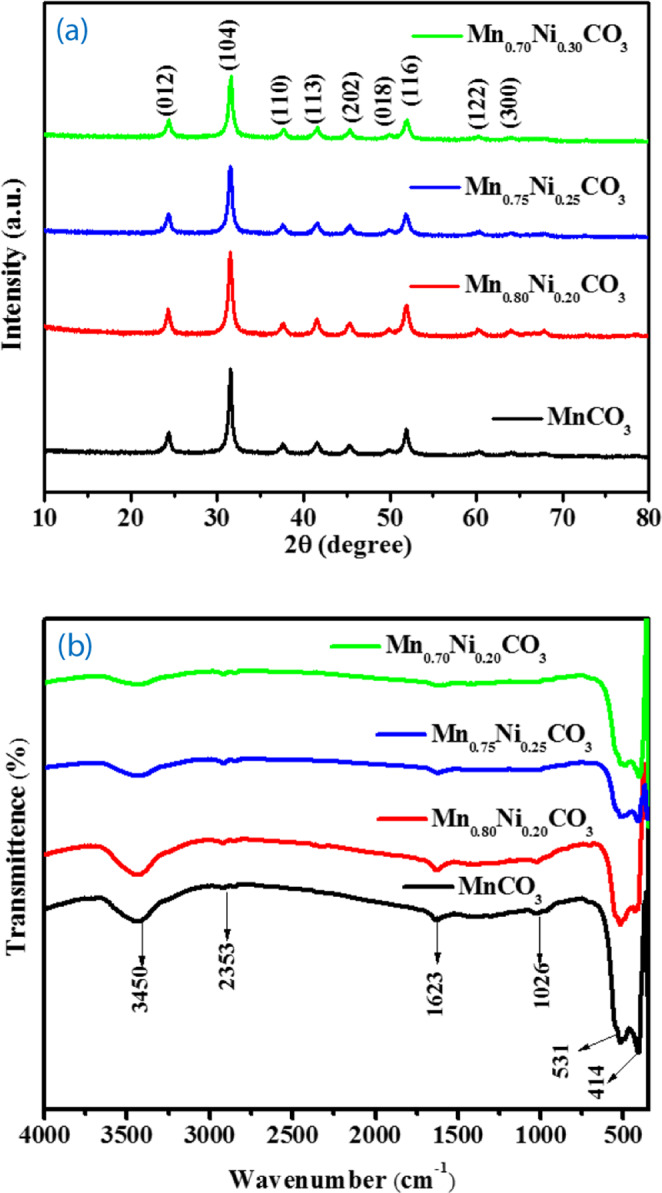
(**a**) XRD patterns and (**b**) FT-IR spectra of the MnCO_3_, Mn_0.80_Ni_0.20_CO_3_, Mn_0.75_Ni_0.25_CO_3_ and Mn_0.70_Ni_0.30_CO_3_ nano/sub-microspheres.

The X-ray diffraction (XRD) analysis was employed for the crystallite and phase structural characterization of the samples. Figure 1a shows the XRD patterns of the as-prepared Mn_1-x_Ni_x_CO_3_ (x = 0.0, 0.20, 0.25 and 0.30) nano/sub-microspheres. As can be seen from the spectra, the MnCO_3_, Mn_0.80_Ni_0.20_Co_3_, Mn_0.75_Ni_0.25_CO_3_ and Mn_0.70_Ni_0.30_CO_3_ nano/sub-microspheres samples show the diffraction peaks of (012), (104), (110), (113), (202), (018), (116), (122) and (300) planes which are corresponding to the reflection located at 2Ɵ values of 24.25, 31.36, 37.52, 41.42, 45.18, 51.48, 51.68, 59.18 and 67.70 respectively^27^. All the diffraction peaks are related to the pure rhombohedral phase of MnCO_3_ structure with the space group of *R3-c* (JCPDS card No. 44-1472) and the peak sharpness and broadness show the highly crystalline and nano/sub-micron nature of the particles. The MnCO_3_, Mn_0.80_Ni_0.20_Co_3_, Mn_0.75_Ni_0.25_CO_3_ and Mn_0.70_Ni_0.30_CO_3_ nano/sub-microspheres samples does not shown any new peak associated with other phases such as Mn(OH)_2_, MnNi(OH)_2_, Ni(OH)_2_, NiCO_3_ or metallic Mn and Ni based phases in the samples. As the Ni content increases, the peak intensity decreases due to the generation of charge imbalance arises by the Ni ratio. The average crystallite size of MnCO_3_, Mn_0.80_Ni_0.20_Co_3_, Mn_0.75_Ni_0.25_CO_3_ and Mn_0.70_Ni_0.30_CO_3_ nano/sub-microspheres samples was calculated by using the Debye-Scherere’s equation which are 70 nm 65 nm 50 nm and 43 nm respectively^28^. The data shows that the presence of Ni ions in Mn_0.80_Ni_0.20_Co_3_, Mn_0.75_Ni_0.25_CO_3_ and Mn_0.70_Ni_0.30_CO_3_ prevented the growth of crystal grains and slows down the motion of a grain boundary due to the interruption on a movement of the grain boundaries by Zener pinning^29^. The smaller crystallite size and phase pure of the Mn_0.75_Ni_0.25_CO_3_ nano/sub-microspheres are expected to offer high electron transport at electrode/electrolyte interface for high power applications.

The Fourier transform infrared spectroscopy (FT-IR) is usually employed as an additional probe to find the organic and inorganic functional species present in the samples. The as-prepare MnCO_3_, Mn_0.80_Ni_0.20_Co_3_, Mn_0.75_Ni_0.25_CO_3_ and Mn_0.70_Ni_0.30_CO_3_ nano/sub-microspheres samples were characterized by the FT-IR spectroscopy in the range of 4000–450 cm^−1^ and the respective spectra are depicted in Figure 1b. The MnCO_3_ nano/sub-microspheres still contain water molecules since OH and CO_2_ molecules have the property of chemisorption on to the MnCO_3_ surface when they are exposed to the atmospheres. The Mn_1-__x_Ni_x_CO_3_ samples displayed a broad peak at ~3450 cm^−1^, which is attributed to the O-H stretching vibration and a band at ~1623 represents the bending vibration of water molecules present in the Mn_1-__x_Ni_x_CO_3_ nano/sub-microspheres. These two vibrational modes show the residual water and hydroxyl groups on the nano/sub-microspheres. The broadness of the peak at 3458 cm^−1^ is decreased slowly on Ni indicating the water molecules adsorption diminished. The band in the range of 450–750 cm^−1^ is attributed to the MnCO_3_ rhombohedral sites; MnCO_3_ stretching vibration peak appeared at ~530 is the characteristic peak for the Mn-C-O (MnCO-Mn^2+^ with CO_3_^2−^) and the other peaks at 1026 cm^−1^ is attributed to the C-O stretching vibration of CO_3_^2−^ ion and the 2353 cm^−1^ peak is related to carbon dioxide^30,31^. The intensity of characteristic peak for the Mn-C-O appeared at ~530 cm^−1^ is increasing on the addition of 20% Ni, then on the 25% addition, the peak stabilized and the other peak at 414 cm^−1^ is decreasing and increasing by the consequent additions, these characteristics are the indication of the of the formation of Mn_0.75_Ni_0.25_CO_3_ homogenously. These FT-IR spectra as well as the XRD patterns indicate that the framework and Ni does not interfere with the MnCO_3_ structure.

**Figure 2:**
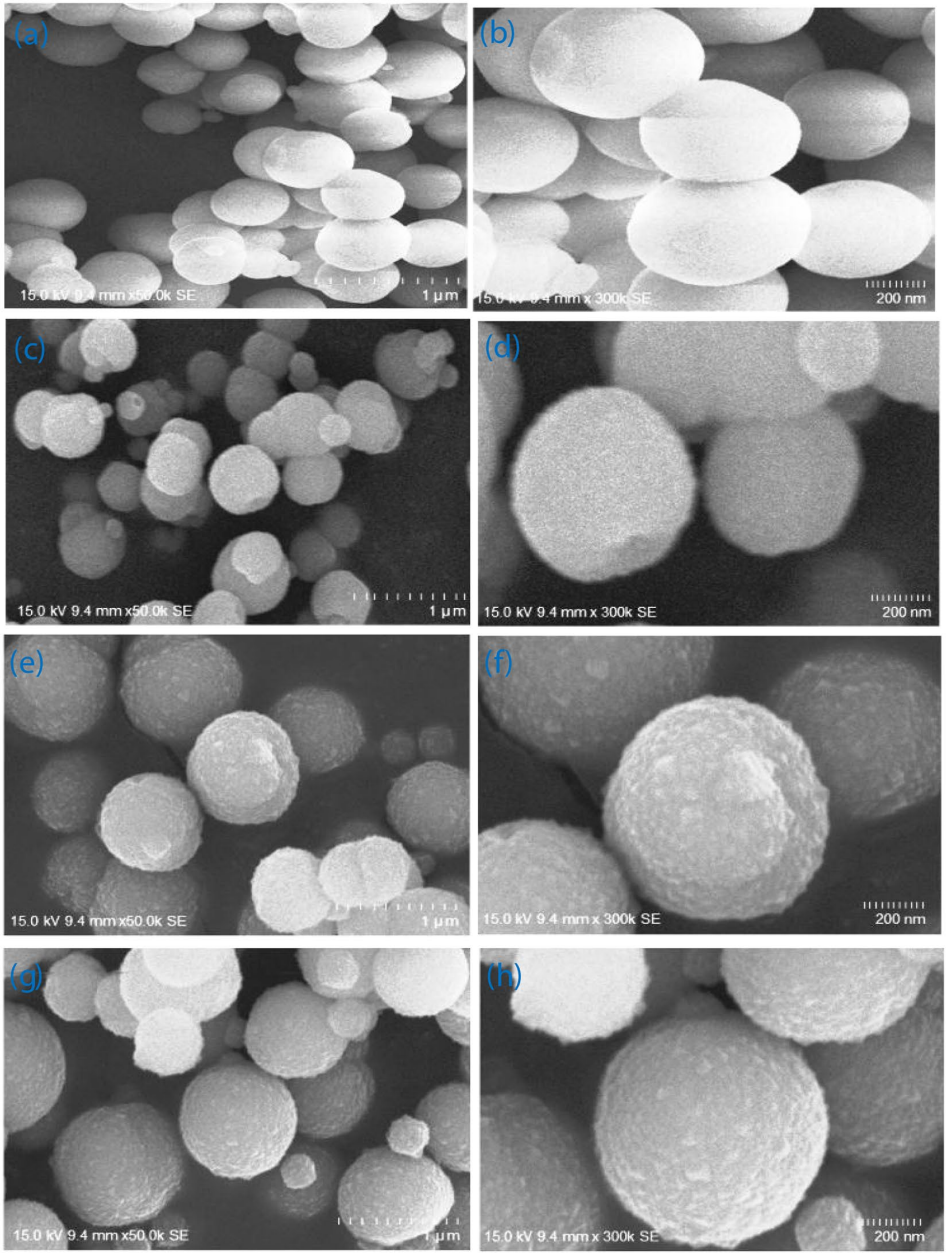
SEM images of (**a, b**) MnCO_3_, (**c, d**) Mn_0.80_Ni_0.20_CO_3_, (**e, f**) Mn_0.75_Ni_0.25_CO_3_ and (**g, h**) Mn_0.70_Ni_0.30_CO_3_ nano/sub-microspheres at low and high-magnifications.

The morphology of the as-prepared MnCO_3_, Mn_0.80_Ni_0.20_Co_3_, Mn_0.75_Ni_0.25_CO_3_ and Mn_0.70_Ni_0.30_CO_3_ nano/sub-microspheres were investigated by scanning electron microscopy presented in Figure 2(a-h). The MnCO_3_, Mn_0.80_Ni_0.20_Co_3_, Mn_0.75_Ni_0.25_CO_3_ and Mn_0.70_Ni_0.30_CO_3_ nano/sub-microspheres are highly uniform and homogenously distributed with an average diameter size of 430 to 470 nm. The formation of nano/sub-microspheres morphology is possible in the co-precipitation method as it takes place in the carbonate medium (digestion of carbonate solution with a base and the precipitation process simultaneously). Under these circumstances, the carbonate solution controls the particles agglomeration and leads to the formation of thermodynamically stable sphere shape of MnCO_3_, Mn_0.80_Ni_0.20_Co_3_, Mn_0.75_Ni_0.25_CO_3_ and Mn_0.70_Ni_0.30_CO_3_ nano/sub-microspheres. The smaller Ni^2+^ ion (70 pm) with the bigger (81 pm) Mn^2+^ ion lattice, contracts the crystals, thus smaller crystallites are formed XRD, FT-IR and SEM results are corroborated one another. Surface roughness is also increases with increasing the Ni^2+^ level, which attracts more electrolyte and expected higher capacitance and faster exchange of e^-^ on its surface.

**Figure 3:**
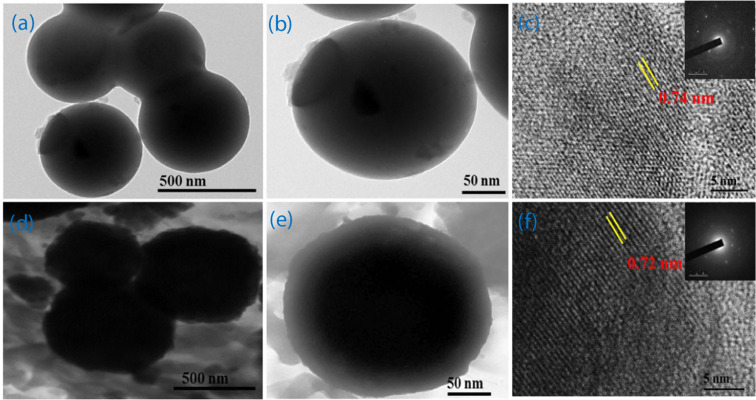
TEM images of (**a–c**) MnCO_3_, (**d–f**) Mn_0.75_Ni_0.25_CO_3_ nano/sub-microspheres at low and high-magnifications.

The TEM micrographs for MnCO_3_ and Mn_0.75_Ni_0.25_CO_3_ nano/sub-microspheres at different magnifications are shown in Figure 3(a-f) and these images are in moral agreement with the SEM micrographs in terms of the nanospheres morphology and aggregation nature. The sizes of the nano/sub-microspheres are 430 nm (MnCO_3_) and 450 nm (Mn_0.75_Ni_0.25_CO_3_). Figure 3c,f show the lattice fringes of MnCO_3_ and Mn_0.75_Ni_0.25_CO_3_ nano/sub-microspheres, demonstrate that the lattice distance is 7.4 Å (MnCO_3_) and 7.2 Å (Mn_0.75_Ni_0.25_CO_3_) agrees well with (104) planes and the inset images of both are the MnCO_3_ and Mn_0.75_Ni_0.25_CO_3_ well well-defined spots arranged in circular rings confirms the poly crystalline formations. Both the nanosphere surface possesses pores, which can offer an effective electron and ion transport consequently supports for the improved electrochemical performance for the supercapacitor applications.

**Figure 4:**
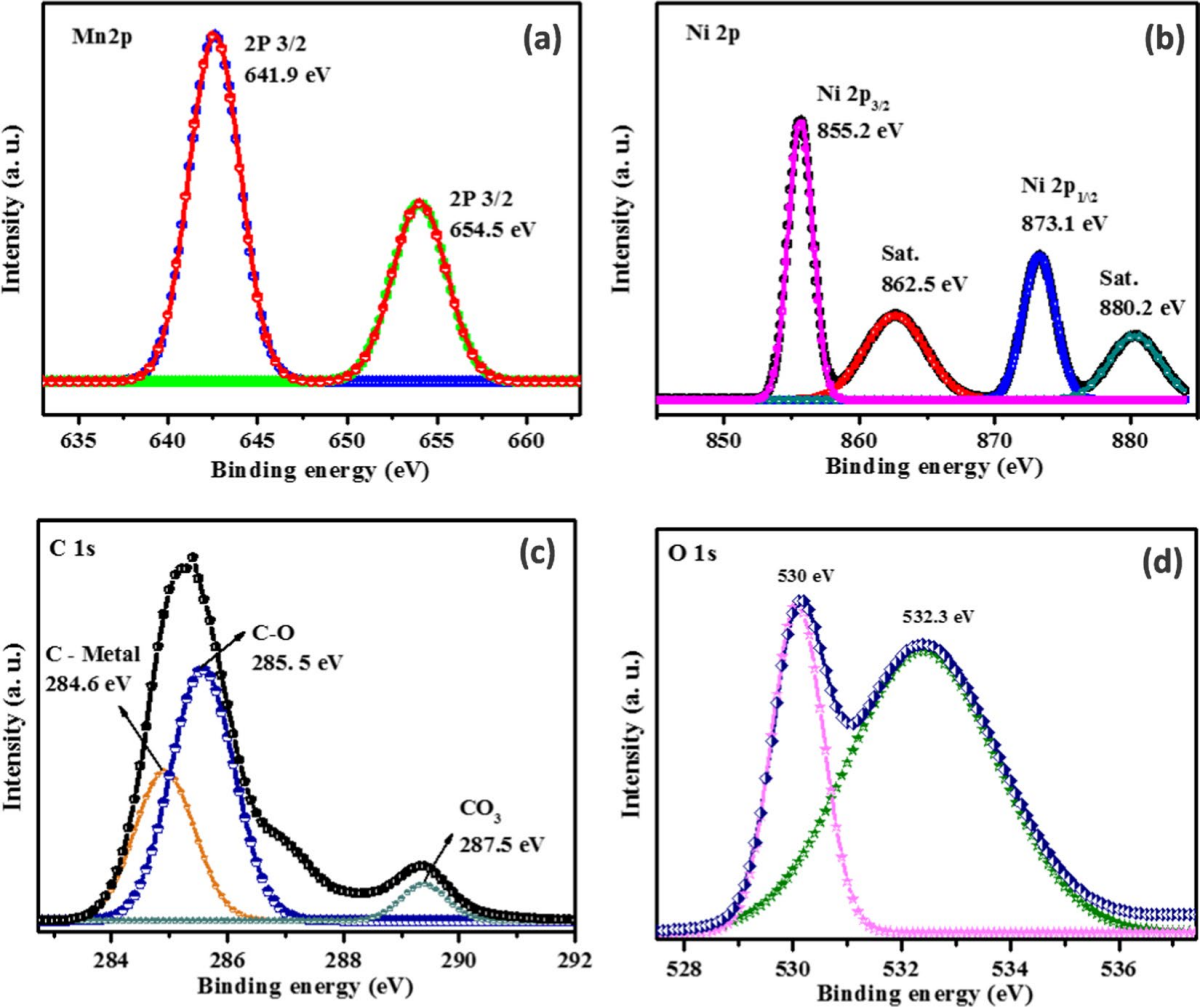
High-resolution XPS spectrum of the Mn_0.75_Ni_0.25_CO_3_ nano/sub-microspheres (**a**) Mn2p spectrum, (**b**) Ni 2 P spectrum, (**c**) deconvoluted C1s spectrum and (**d**) O 1s spectrum.

Additionally, the elemental chemical composition and electronic state of the Mn_0.75_Ni_0.25_CO_3_ was determined using X-ray photoelectron spectroscopy (XPS) technique shown in Figure 4(a–d) and the survey spectrum is provided in Figure S1 (representative). As shown in Figure 4a, the binding energy peaks at 641.9 eV and 654.5 eV are assigned to Mn 2p_3/2_ and Mn 2p_1/2_ respectively, which coincide with the Mn^2+^ state of Mn in the Mn_0.75_Ni_0.25_CO_3_ samples^32,33^. The convolution of Ni 2p peaks at binding energy positions of 855.2 eV and 873.1 eV attributed to the Ni 2p_3/2_ and Ni 2p_1/2_ respectively and other two satellite (shake-up process) peaks appeared at 862.5 eV and 880.2 eV are correspond to the Ni 2p_3/2_ and Ni 2p_1/2_ respectively, as shown in Figure 4b. The main peaks and satellite peaks emerged for the Ni 2p region are owing to the presence of Ni^2+^ state in the Mn_0.75_Ni_0.25_CO_3_ samples^34,35^. The high resolution spectra of C 1 s was convolute into three binding energy peaks and shown in Figure 4c. Three main binding energy peaks at 284.6 eV, 285.5 eV and 287.5 eV can be assigned to the characteristics bands of C-Mn, C-O and C-OO, respectively, indicating the presence of carbonate^36–38^. The O 1 s spectra (Figure 4d) show that the peak appeared at 532.3 eV is ascribed to surface adsorption of the material oxygen and the other peak at 530.0 eV is typical characteristic peaks of the metal – oxygen bonds^32–34^. Thus, the XPS data revealed the presence of Mn, Ni, O and C without other impurity elements, which is consistent with the XRD results.

## Electrochemical Characterization

**Figure 5:**
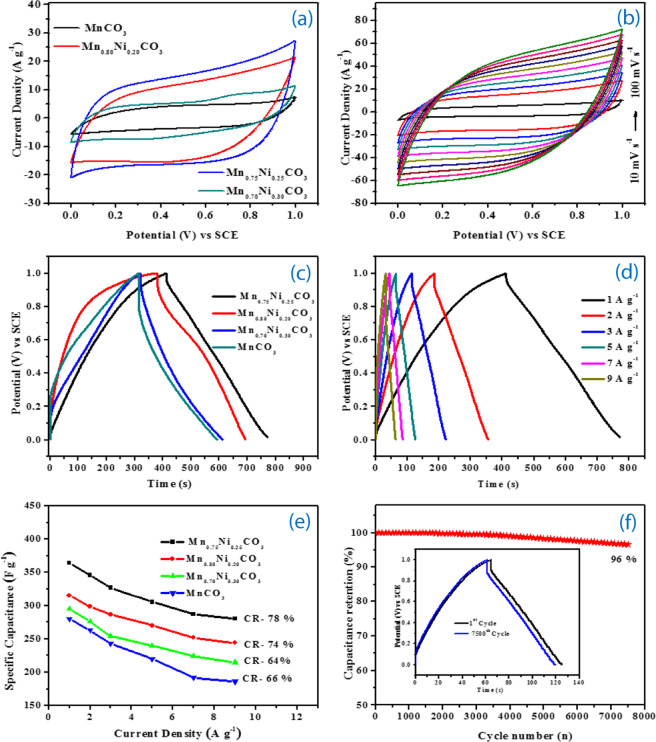
Electrochemical performance of the synthesized pristine MnCO_3_, Mn_0.80_Ni_0.20_CO_3_, Mn_0.75_Ni_0.25_CO_3_ and Mn_0.70_Ni_0.30_CO_3_ nano/sub-microspheres electrode samples in 3-electrode cell: (**a**) Comparison CV curves at 20 mV s^−1^, (**b**) CV curves of the Mn_0.75_Ni_0.25_CO_3_ nano/sub-microspheres electrode at diverse scanning rates (10 to 100 mV s^−1^) between 0.0 and 1 V, (**c**) Comparison GCD curves of the nano/sub-microspheres electrodes at 1 A g^−1^, (**d**) GCD curves for the Mn_0.75_Ni_0.25_CO_3_ nano/sub-microspheres electrode at diverse current density (1 to 9 A g^−1^), (**e**) specific capacitance of the nano/sub-microspheres electrodes at various current densities and (**f**) cycling stability of the Mn_0.75_Ni_0.25_CO_3_ nano/sub-microspheres measured at 5 A g^−1^ for 7500 cycles, 1^st^ and 7500 charge-discharge cycles (insert)_._.

The electrochemical performances of the as-prepared MnCO_3_, Mn_0.80_Ni_0.20_Co_3_, Mn_0.75_Ni_0.25_CO_3_ and Mn_0.70_Ni_0.30_CO_3_ nano/sub-microspheres electrodes were investigated in a three-electrode system. Figure 5a compares the cyclic voltammetry (CV) curves of various electrodes tested at a scan rate of 20 mV s^−1^ with the potential window of 0.0 V–1.0 V Vs SCE in 1 M of Na_2_SO_4_ electrolyte. These CV curves are quasi-rectangular shape, indicating an ideal electrical double layer capacitance with no O_2_ or H_2_ gas evolution peaks and the area of the Mn_0.75_Ni_0.25_CO_3_ nano/sub-microspheres electrode are larger than that of the other nanospheres electrodes. The superior electrochemical property of Mn_0.75_Ni_0.25_CO_3_ nano/sub-microspheres electrode is due to the synergistic effects of Ni and Mn elements in the Ni-Mn-CO_3_ solid solution with proper Ni ratio spherical morphology and smaller crystallites size with pores natures. Hence, CV curves of the Mn_0.75_Ni_0.25_CO_3_ nano/sub-microspheres electrode measured at diverse scan rates ranging from 10 mV s^−1^ to 100 mV s^−1^ are measured and illustrated in Figure 5b and for the other three electrodes presented in Figure S2. Thus, denote that the shapes of the CV curves are retained well as the scan rate increases, indicating rapid electronic and ionic transportation at the electrode/electrolyte interface. The charge storage mechanism of Mn_0.75_Ni_0.25_CO_3_ is explained based on the intercalation/de-intercalation mechanism as follows:

1 $${{\rm{Mn}}}_{0.75}{{\rm{Ni}}}_{0.25}{{\rm{CO}}}_{3}+{{\rm{Na}}}^{+}+{{\rm{e}}}^{-}\leftrightarrow {{\rm{NaMn}}}_{0.75}{{\rm{Ni}}}_{0.25}{{\rm{CO}}}_{3}$$

During the charging process, the Na^+^ ions from the electrolyte intercalates into Mn_0.75_Ni_0.25_CO_3_ matrix and release one electron. On the other hand, during discharging Na^+^ ions are de-intercalated from Mn_0.75_Ni_0.25_CO_3_ matrix and diffuse into the electrolytic solution.

Galvanostatic charge/discharge (GCD) measurements are further evaluated in the potential window range between 0.0 V and 1.0 V to assess the performance of various nano/sub-microspheres electrodes at 1 A g^−1^ (Figure 5c). The superior performance was obtained for the Mn_0.75_Ni_0.25_CO_3_ nano/sub-microspheres electrode and interrogated at various current densities from 1 A g^−1^ to 9 A g^−1^ (Figure 5d). The charge/discharge curves for MnCO_3_, Mn_0.80_Ni_0.20_Co_3_, and Mn_0.70_Ni_0.30_CO_3_ electrodes are presented in Figure S3. The specific capacitance of the various nano/sub-microspheres electrodes is calculated by using the following formula,

2 $${{\bf{C}}}_{{\bf{s}}}=\frac{i\Delta t}{m\Delta v}$$

Where, Cs is specific capacitance (F g^−1^), *i* is the constant current (A), *∆t* is the discharge time (s), m is the total mass of the active material, and *∆v* is the potential window (V)^39^. In our charge - discharge studies, the specific capacitance contribution from the nickel foam current collector was ignored. The specific capacity value of MnCO_3,_ Mn_0.80_Ni_0.20_CO_3_, Mn_0.75_Ni_0.25_CO_3_ and Mn_0.70_Ni_0.30_CO_3_ nano/sub-microspheres electrodes is 280, 295, 364 and 315 F g^−1^ respectively at 1 A g^−1^. Higher specific capacitance value was obtained for Mn_0.75_Ni_0.25_CO_3_ electrode owing to its higher surface area and stabilized structure. Whereas, at 30% Ni, disintegration (Mn_0.70_Ni_0.30_CO_3_) of crystal structure reduces the capacitance. The specific capacitance of Mn_1-__x_Ni_x_CO_3_ (x-0, 0.20, 0.25 and 0.30) nano/sub-microspheres electrodes with different current densities are presented in the Figure 5e. Among the all samples, Mn_0.75_Ni_0.25_CO_3_ exhibits higher specific capacitance than the other fabricated electrodes in this work and also with Mn_0.75_Ni_0.25_CO_3_ previously reported values (Table 1).

**Table 1:** Compression of specific capacitance and capacity retention with other Mn-based materials reported in the literature.

Sample Preparation Method	Material Name	Electrolyte	Potential window	Specific capacitance (F g^−1^)	Capacity retention	Ref. No.
SILAR	MnCO_3_-RGO	1 M Na_2_SO_4_	0.0 V–0.8 V	157	87% at 1000 cycle	^23^
Self-assembly method	MnCO_3_@MnO_2_	1 M Na_2_SO_4_	−0.2 V–0.8 V	363	—	^24^
Hydrothermal	NiFeO_x_@MnCO_3_	3 M KOH	0.0 V–1.0 V	283	92.7% at 2000 cycles	^25^
Hydrothermal	MnCO_3_	0.1 M Na_2_SO_4_	0.0 V–1.0 V	216	97% at 500 cycle	^26^
Precipitation	MnO_2_	6.0 M KOH	0.0 V–0.9 V	193	94% at 1300 cycle	^43^
Hydrothermal	Mn_3_O_4_	1 M Na_2_SO_4_	−0.1 V–0.7 V	114	100% at 1000 cycles	^44^
Precipitation	Mn_3_O_4_	1 M Na_2_SO_4_	−0.1 V–0.8 V	121	100% at 1400 cycle	^31^
Solvothermal	Mn_3_O_4_	1 M Na_2_SO_4_	−0.8–0.6 V	131	99% at 500 cycle	^45^
Hydrothermal	MnO_2_	0.5 M Na_2_SO_4_	0.0 V–1.0 V	322	86% at 2000 cycle	^46^
**Co-precipitation**	**Mn**_**0.75**_**Ni**_**0.25**_**CO**_**3**_	**1 M Na**_**2**_**SO**_**4**_	**0.0 V**–**1.0 V**	**364**	**96% at 7500 cycles**	**This work**

The specific capacitance of the Mn_0.75_Ni_0.25_CO_3_ nano/sub-microspheres electrode (364 F g^−1^; 23.07% %) is higher than that of pristine MnCO_3_ (280 F g^−1^ at 1 A g^−1^) in 1 M of Na_2_SO_4_ electrolyte. The specific capacity retention of the Mn_0.75_Ni_0.25_CO_3_ is slowly decreased (280 F g^−1^) by increasing the current density and retained about 78% at 9 A g^−1^ current density. Whereas, the MnCO_3_ nano/sub-microspheres electrode exhibits only 66% specific capacitance retention of at 9 A g^−1^. The specific capacitance of Mn based metal oxides electrodes are shown in Table 1. The significant specific capacitance retention is offered only by the Mn_0.75_Ni_0.25_CO_3_ nano/sub-microspheres.

The practical performance is highly important for the application of any energy systems, particularly in hybrid electric vehicles and renewable energy storage systems. Hence, the long-term cycle stability studies for the Mn_0.75_Ni_0.25_CO_3_ nano/sub-microspheres electrode was measured over 7500 continuous charge-discharge cycles at a specific current of 5 A g^−1^ in 1 M of Na_2_SO_4_ electrolyte (Figure 5f) and it retains about 96% capacitance retention even after 7500 cycles. Within the test voltage window, the intercalation and de-intercalation processes of the guest ions are taken place significantly structural in the meso-structural electrodes. Further, there were no structural changes in the electrode observed, as it showed very stable cycle life.

The electrochemical impedance spectra (EIS) of the MnCO_3,_ Mn_0.80_Ni_0.20_CO_3_, Mn_0.75_Ni_0.25_CO_3_ and Mn_0.70_Ni_0.30_CO_3_ nano/sub-microspheres electrodes were taken in the frequency range from 100 mHz to 100 kHz and the results are shown in Figure S4. The Nyquist plot of nano/sub-microspheres electrode showed a semicircle at the high frequency region and a straight line at the low frequency region. An equivalent circuit was fitted by using Zview software and the charge-transfer resistance (*R*_*ct*_) of the MnCO_3,_ Mn_0.80_Ni_0.20_CO_3_, Mn_0.75_Ni_0.25_CO_3_ and Mn_0.70_Ni_0.30_CO_3_ nano/sub-microspheres electrodes is 17.59, 6.45, 5.51 and 9.81 ohm, respectively. Evidently, the lower resistance value is obtained for Mn_0.75_Ni_0.25_CO_3_ nano/sub-microspheres electrode, which result better electrochemical performance. The fast transfer of charged species between the electrode and electrolyte is confirmed by the lower R_ct_ values of the Mn_0.75_Ni_0.25_CO_3_ nano/sub-microspheres electrode, which could lead to good electrochemical performance. The synergism between Ni and Mn give rise to good specific capacitance and cycle life^25,40^.

### Electrochemical performance of solid-state asymmetric supercapacitor

**Figure 6:**
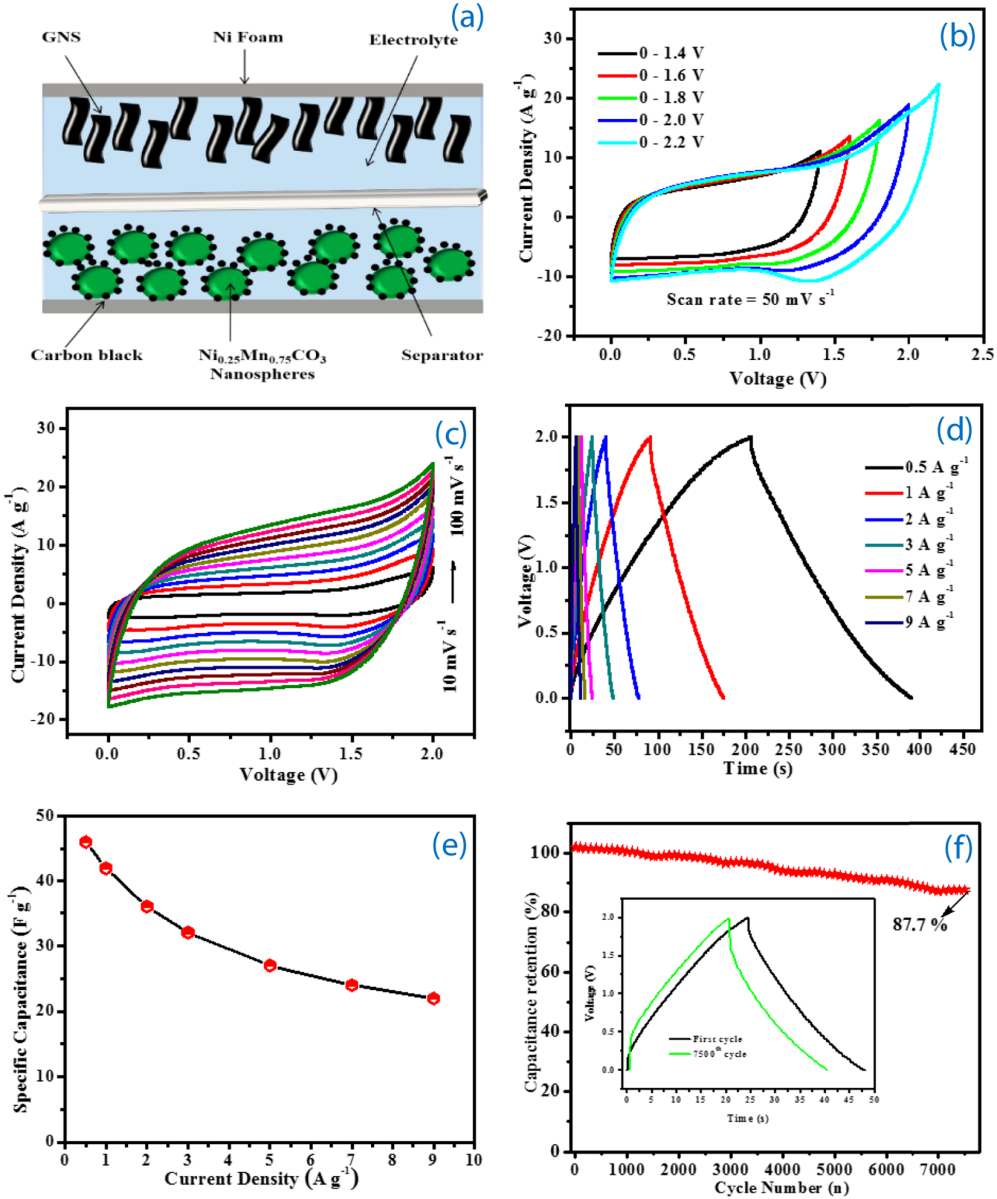
(**a**) Schematic illustration of the solid-state asymmetric supercapacitor configuration, (**b**) CV curves of Mn_0.75_Ni_0.25_CO_3_//GNS solid-state ASC measured at different potential window at a scan rate of 50 mV s^−1^, (**c**) CV curves of the solid-state ASC measured at different scan rates (10 to 100 mV s^−1^), (**d**) GCD curves at different current densities (0.5 to 9 A g^−1^), (**e**) Calculated specific capacitance of solid-state ASC at different current density (0.5–9 A g^−1^), (**f**) long-term cyclic stability of solid-state ASC over 7500 cycles at 3 A g^−1^, 1^st^ and 7500 charge-discharge cycles (insert)_._.

To further evaluate the practical applicability of the Mn_0.75_Ni_0.25_CO_3_ nano/sub-microspheres, we assembled solid-state ASC device using Mn_0.75_Ni_0.25_CO_3_ nano/sub-microspheres as the positive electrode, graphene nanosheets as the negative electrode and PVA- Na_2_SO_4_ as the solid-state electrolyte (Figure 6a). For the fabrication of solid-state ASC, GNS was used as negative electrode, its phase and morphology were examined (Figure S5) and the specific capacitance of GNS is 115 F g^−1^ at 1 A g^−1^, calculated from charge discharge curves in Figure S6a. Figure S6b shows, the CV curves of GNS and Mn_0.75_Ni_0.25_CO_3_ nano/sub-microspheres electrodes, whose stable voltage windows were identified as −1.0 V to 0.0 V and 0.0 V to 1.0 V, respectively. The solid-state ASC device exhibits a capacitive behaviour with almost rectangular CV curves with no obvious redox peak at various operating potential window between 0.0 V and 2.2 V at 50 mV s^−1^, resulting spectra are presented in Figure 6b. The effect of scan rates was evaluated for a solid-state ASC device in the voltage window from 0 to 2.0 V at various scan rates from 10 to 100 mV s^−1^ and presented in Figure 6c. The ASC device CV profile of cell has remained relatively rectangular shape even at a higher scanning rate of 100 mV s^−1^ without any other distraction in the double layer behaviour. This electrochemical characteristic offered by the fabricated electrode/electrolyte interfacial phenomenon are expected to offer good charge / discharge properties with higher rate capability characteristics.

**Figure 7:**
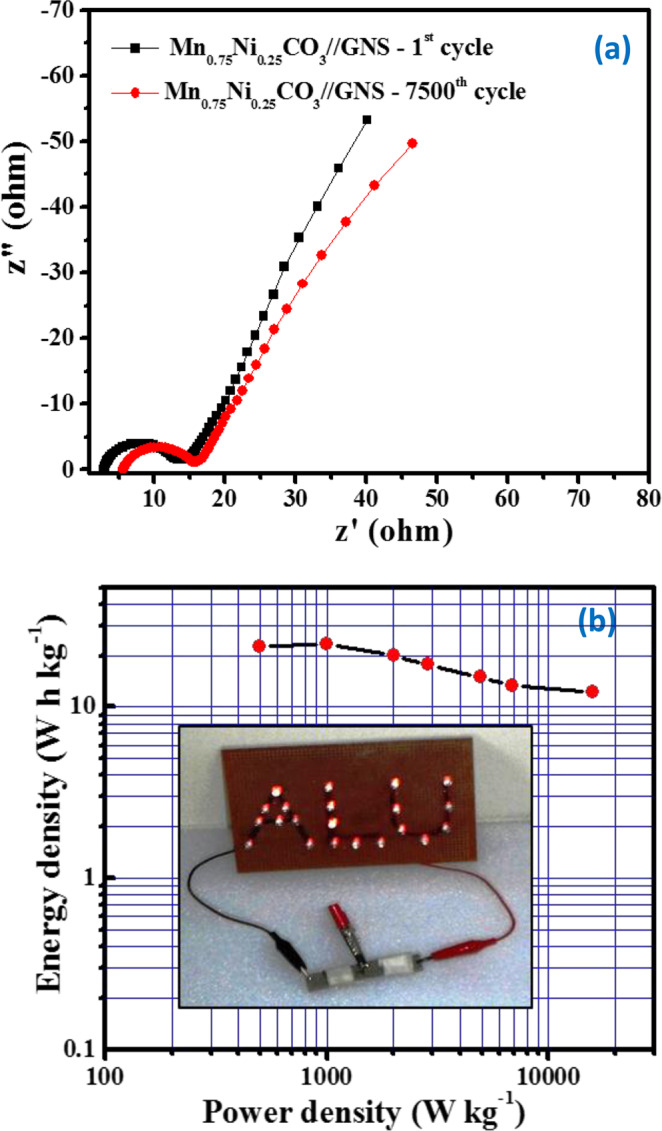
(**a**) EIS curves of the Mn_0.75_Ni_0.25_CO_3_ nano/sub-microspheres//GNS solid-state ASC initial cycles and after 7500 cycles, (**b**) Ragone plots of the Mn_0.75_Ni_0.25_CO_3_ nano/sub-microspheres//GNS solid-state ASC, lighted LED (insert).

The galvanostatic charge-discharge (GCD) curves at different current densities are shown in Figure 6d and it can be seen that the potential of the charge-discharge profile indicates the higher discharge time. The discharge curves at different current density of 0.5, 1.0, 2.0, 3.0, 5.0, 7.0 and 9.0 A g^−1^, are providing the specific capacitance of 46, 42, 36, 32, 27, 24 and 22 F g^−1^, respectively (Figure 6e). Furthermore, 87.7% capacitance retention was achieved even after 7500 charge-discharge cycles at the higher current density of 3 A g^−1^ (Figure 6f), testifying its high rate capability. This capacity retention capability is possibly due to synergistic effect mainly related to the stable nano/sub-microspheres morphology and structure of electrode materials build during the synthesis of the precursor by sodium bicarbonate, where the nanoparticles surface roughness provides more active surface area, less resistance for electron/ion transport at the electrode/electrolyte interface and stable phase structure. The electrochemical impedance spectra were taken for the ASC device before and after 7500 cycles, there was negligible variation in the charge transfer resistance and solution resistance values was noted, due to the stable electrode and electrolyte configurations, as shown in Figure 7a. Herein, we have provided the energy density and power density of Mn_0.75_Ni_0.25_CO_3_//GNS ASC in the Ragone plot (Figure 7b), these two parameters characterize the performance of a ASC, which can be calculated using the following equations:

3 $${\rm{E}}=[{\rm{Cs}}\times {(\Delta V)}^{2}]/2\times 3.6$$

4 $${\rm{P}}={\rm{E}}\times 3600/\Delta {\rm{t}}$$

Where E, Cs, ∆V, P and ∆t are the energy density (Wh kg^−1^), specific capacitance (F g^−1^), discharge potential (V), power density (kW kg^−1^) and discharge time (s), respectively^23,41^. The energy density of the fabricated ASC decreases from 25 to 12.22 W h kg^−1^ as the power density increases from 499 to 15840 W kg^−1^ (The ASC device of Mn_0.75_Ni_0.25_CO_3_//GNS can be used to light the red-light emitting diode (LED) after being charged (insert Figure 7b)). Nanostructured Mn_0.75_Ni_0.25_CO_3_ nano/sub-microspheres solid-state ASC exhibits superior electrochemical performance; due to their definite size effect, high surface area, low density, and permeation of Na^+^ ion in the Mn_0.75_Ni_0.25_CO_3_ nano/sub-microspheres electrodes. There are few literature reports on the fabrication of Mn based ASC electrode materials available and their performance are compared (Table 2). Most importantly, at lower current density of 0.5 A g^−1^, 499 W kg^−1^ power density was observed and at higher current density of 9 A g^−1^, 15540 W kg^−1^ power density was obtained, this value is enough to meet the power demands for the PNGV (Partnership for a New Generation of Vehicles). These results reveal that our Mn_0.75_Ni_0.25_CO_3_//GNS solid-state ASC device possess a great potential for practical applications. The high energy and power density of solid-state ASC applications electrode materials are due to the uniform nano/sub-microspheres morphology as cathode and more surface area of GNS as an anode material that lead to more accessible to smaller electrolyte ions and favourable for the fast diffusion of the electrolyte ions at the surface of electrode materials. Meanwhile, the solid-state ASC electrode materials prevent the loss of capacity retention during the repetitive incorporation/extraction process. The highlighted solid-state ASC device (Mn_0.75_Ni_0.25_CO_3_//GNS) is novel, cost effective, easy to fabricate and possesses an excellent potential for energy and power density devices.

**Table 2:** Compression of energy density, power density, potential window and specific capacitance previous literatures work on Mn based asymmetric supercapacitors.

Material Name	Specific capacitance	Potential window	Current density	Energy density	Power density	References
MnCO_3_@MnO_2_	60.8 F g^−1^	1.8 V	0.3 A g^−1^	27.4 3 Wh kg^-^	271.7 W kg^−1^	^24^
MnO_2_//AG	50 F g^−1^	1.0 V	0.25 A g^−1^	22.5 Wh kg^−1^	146.2 kW kg^−1^	^47^
MnO_2_/La_2_O_3_//AC	46 F g^−1^	2.0 V	0.3 A g^−1^	25.8 W h kg^−1^	0.3 W kg^−1^	^48^
PM//HMC	48 F g^−1^	1.6 V	1.08 mA g^−1^	14.7 Wh kg^−1^	90 W kg^−1^	^49^
CNFs//MnO_2_	45 F g^−1^	1.8 V	0.5 A g^−1^	20.3 Wh kg^−1^	485 W kg^−1^	^50^
Mn_0.75_Ni_0.25_CO_3_ // GNS	46 F g^−1^	2.0 V	0.5 A g^−1^	25 Wh kg^−1^	499 W kg^−1^	This work

## Conclusions

In conclusion, we have reported the facile template-free synthesis of Mn_1-_xNi_x_CO_3_ (x-0, 0.20,0.25 and 0.30) nano/sub-microspheres *via* facile co-precipitation method using sodium bicarbonate as the precipitating agent for the first time. The microscopic and X-ray diffraction results reveal that the synthesized rhombohedral Mn_1-_xNi_x_CO_3_ particles are homogeneously dispersed crystalline nano/sub-microspheres morphologies with 430–470 nm size. Galvanostatic charge-discharge result showed high specific capacity of 364 F g^−1^ offered by Mn_0.75_Ni_0.25_CO_3_ nano/sub-microspheres electrode which is 23.07% higher capacity than the capacity offered by MnCO_3_ in 1 M of Na_2_SO_4_ electrolyte. The Mn_0.75_Ni_0.25_CO_3_ nano/sub-microspheres electrode is to be one of the promising electrodes for long-term cycle stability as it exhibited stabilized performance at 5 A g^−1^ for 7500 cycles. Further, the as-fabricated Mn_0.75_Ni_0.25_CO_3_//GNS device displayed a power density of 499 W kg^−1^ at high specific energy density of 25 Wh kg^−1^, as well as the solid-state ASC capacitance retention of 87.7% was delivered even after 7500 cycles, which is an additional benefit, and suitable for the commercial applications. The Mn_0.75_Ni_0.25_CO_3_ nano/sub-microspheres materials further provides a new pathway for the new Li-ion anode, sensor and photocatalyst applications.

## Experimental

### Materials

Nickel sulphate hexahydrate (NiSO_4_.6H_2_O), Manganese sulphate tetrahydrate (MnSO_4_.4H_2_O), sodium bicarbonate (NaHCO_3_), Sodium sulfate (Na_2_SO_4_), Acetylene black, Polyvinylidene fluoride and N-Methyl-2-Pyrrolidone were procured from Sigma-Aldrich, India. Ethanol was purchased from SRL Pvt. Ltd, India. All the purchased chemicals and regents were in analytical grade and used as received without any further purifications.

### Synthesis of Mn_1-x_Ni_x_CO_3_ (x = 0.0, 0.20, 0.25 and 0.30) nano/sub-microspheres

Mn_1-x_Ni_x_CO_3_ nano/sub-microspheres were synthesized by a facile co-precipitation method. Typically, manganese sulphate (0.75 mmol) and nickel sulphate (0.25 mmol) were dissolved in double distiller water (70 mL) and sodium bicarbonate (10 mmol) was dissolved separately in double distiller water (70 mL). A 7 mL of ethanol was then added to the MnSO_4_ and NiSO_4_ solutions with constant stirring. After its complete dispersion, the NaHCO_3_ solution was added to the above mixture at room temperature. After 5 min, the reaction solution was turned to green colour. This indicated that the initial formation of Mn_0.75_Ni_0.25_CO_3_ nano/sub-microspheres and mixture was to continuously stirred for 3 hours at room temperature to form Mn_0.75_Ni_0.25_CO_3_ nano/sub-microspheres. The Mn_0.75_Ni_0.25_CO_3_ nano/sub-microspheres formed were separated by filtration and washed several times with ultrapure water and ethanol to remove impurities. Finally, Mn_0.75_Ni_0.25_CO_3_ nano/sub-microspheres were dried at 120 °C for 12 h to remove the adsorbed water molecules on the surface of the nano/sub-microspheres. The same procedure was followed for the synthesis of Mn_1-x_Ni_x_CO_3_ (x = 0.0, 0.20, 0.25 and 0.30) nano/sub-microspheres.

### Materials characterization

The structural, functional and morphologies of Mn_1-x_Ni_x_CO_3_ nano/sub-microspheres were examined using X-ray diffraction (XRD) measurements using a PAN Analytical X’ per PRO Model X-ray diffractometer with Cu Kα radiation (α = 1.5418 Å) from 10–80 °, Fourier transform infrared (FT-IR) spectral analyses were performed using a Nicolet Avater 370 with KBr pellet technique from 450 to 4000 cm^−1^. The morphology and surface nature of Mn_1-x_Ni_x_CO_3_ nano/sub-microspheres were characterized using scanning electron microscopy using JEOL-JSM and transmission electron microscopy (TEM) taken on a JEOL/JEM 2100. X-ray photoelectron spectroscopy (Carl Zeiss) was carried out in ultra-high vacuum with Al kα line (1486.6 eV) as an exciting source to analyse the surface chemistry and valence state of Mn_0.75_Ni_0.25_CO_3_ sample^20^.

### Electrochemical measurements

To evaluate the electrochemical properties of the synthesized Mn_1-x_Ni_x_CO_3_ (x = 0.0, 0.20, 0.25 and 0.30) nano/sub-microspheres electrodes the cyclic voltammetry (CV), galvanostatic charge-discharge (GCD) and electrochemical impedance spectroscopy studies with three electrode system used. The working electrode was prepared by mixing the synthesized Mn_1-x_Ni_x_CO_3_ nano/sub-microspheres materials (active materials-70%), conductive materials (acetylene black-20%), binder (polyvinylidene fluoride (PVDF-10%)) and a few drops of n-methyl-2-pyrrolidone (NMP) was used as the solvent. The active material (1.5 mg) was coated on a nickel foam substrate (1 cm ×1 cm) and was dried at 90 °C for 10 h. This served as the working electrode, saturated calomel (SCE) and a platinum wire was used as the reference and counter electrode, respectively. The electrode evaluated in 1 M of Na_2_SO_4_ aqueous solutions electrolyte at different scan rate and current densities.

The constructed solid-state ASC device was measured with a two-electrode system, including two slices of nickel foam (2 ×1 cm) as current collectors and a cellulosic paper as a separator. In the two-electrode system, the Mn_0.75_Ni_0.25_CO_3_ nano/sub-microspheres were used as the positive electrode (1.2 mg) and the graphene nanosheet (GNS) (3.87 mg) was used as the negative electrode, which was prepared by pasting of 20% acetylene black and 10% of PVDF in NMP slurry on a nickel foam. The solid-state electrolyte was prepared by the addition of 2 g PVA powder into 20 ml of deionized water under vigorous stirring at 95 °C, a clear solution was obtained. Later, 1 g of Na_2_SO_4_ was added to the above clear solution under stirring for 30 min to form PVA- Na_2_SO_4_ solid -state gel. The positive (Mn_0.75_Ni_0.25_CO_3_ nano/sub-microspheres) and negative (GNS) electrode and separator were dipped into the PVA- Na_2_SO_4_ solid-state solution for 5 min, taken out, assembled and kept under vacuum desicater to form solid-state ASC. For the optimal mass ratio between the positive and negative electrodes (m+/m) was obtained from the maximum capacitance and operating potential window, which was calculated by using the following charge balance equation^42^,

5 $${q}^{+}={q}^{-}$$

6 $$q=m\times c\times v$$

7 $${m}^{+}/{m}^{-}={c}^{-}{v}^{-}/{c}^{+}{v}^{+}$$

Where, *m* is the mass, *c* is the specific capacitance and *v* is the operating voltage range. The electrochemical performances in both configurations such as in three electrode and in the two electrode systems were carried out using the CHI 660D electrochemical work station.

## Supplementary information

Supplementary Information.
