# Supplementary material for: Ultra-stable Mn1-xNixCO3 nano/sub-microspheres positive electrodes for high-performance solid-state asymmetric supercapacitors
Source: Sci Rep. 2020 Jun 1;10:8871. doi: 10.1038/s41598-020-64867-8 (PMC7264220; doi:10.1038/s41598-020-64867-8)
Supplement: Supplementary file 1 — Supplementary Information. [file 41598_2020_64867_MOESM1_ESM.pdf]

Supplementary Information

Ultra-stable $Mn_{1-x}Ni_xCO_3$ nano/sub-microspheres positive electrodes for high-performance solid-state asymmetric supercapacitors

*Srinivasan Alagar, Rajesh Madhuvilakku, and Shakkthivel Piraman**

Sustainable Energy and Smart Materials Research Lab,

Department of Nanoscience and Technology,

Science Campus, Alagappa University,

Karaikudi-630 002, Tamil Nadu, India.

* Corresponding Author

Email: apsakthivel@yahoo.com (Shakkthivel Piraman)

Tel: +91 4565- 223 372; Fax: +91 4565- 225202, 225525

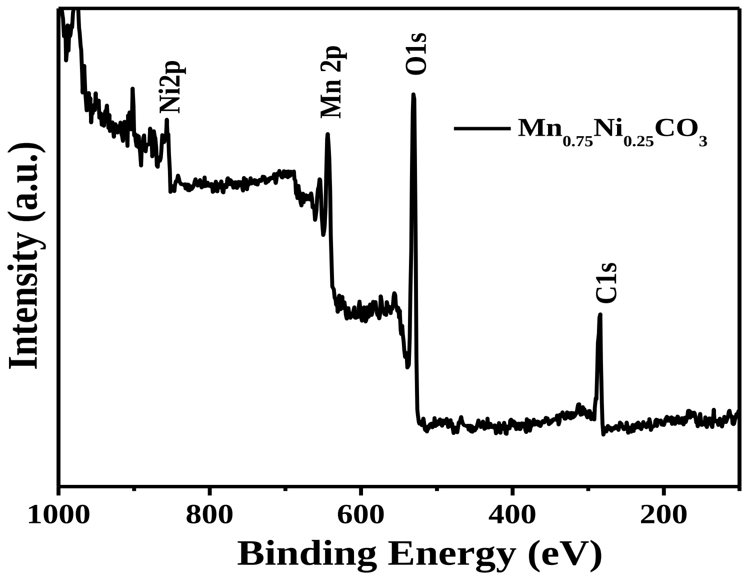

Figure S1. Representative XPS spectra of the $Mn_{0.75}Ni_{0.25}CO_3$ sample wide spectrum.

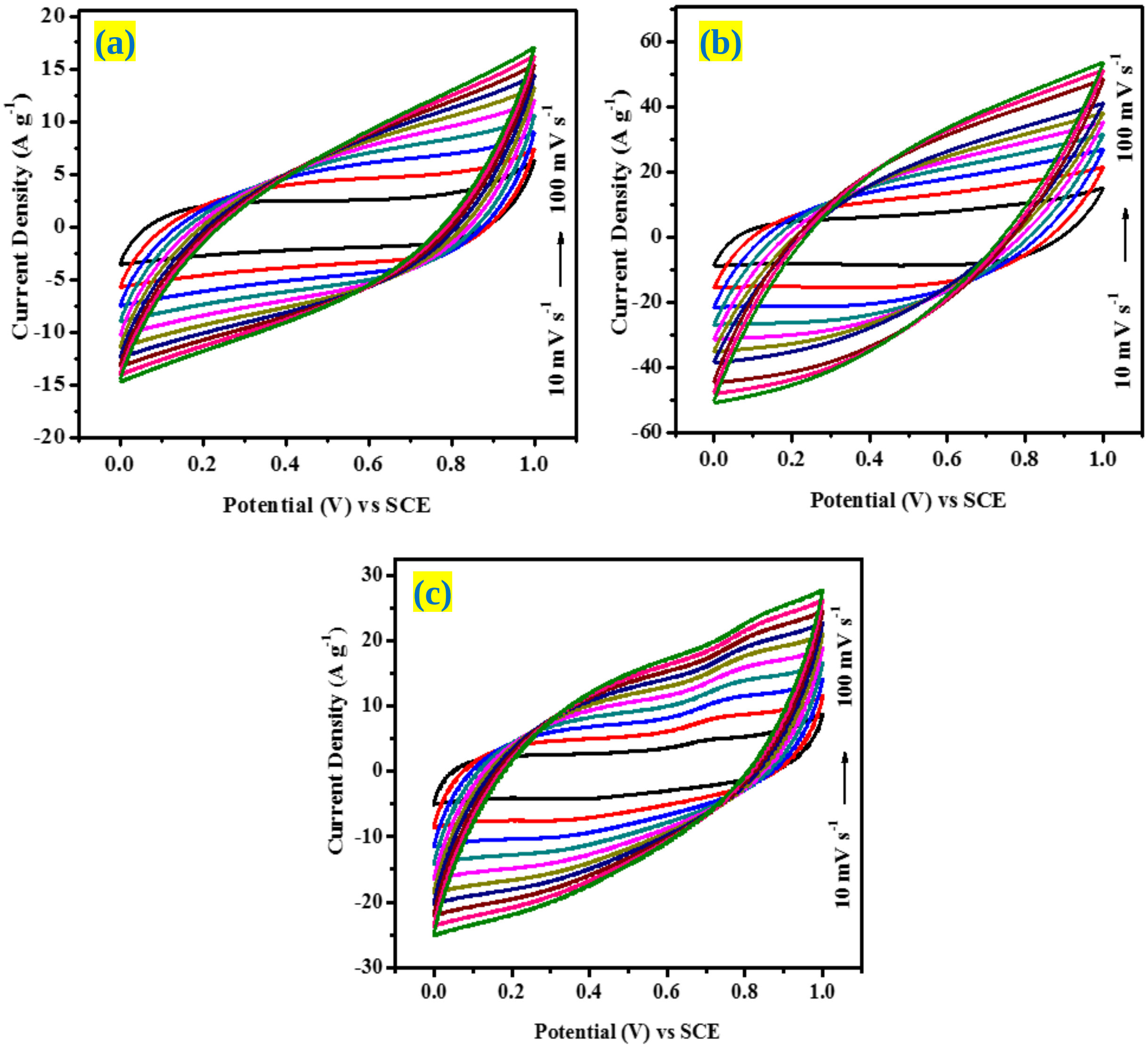

Figure S2. Electrochemical performance of the synthesized samples at 3-electrode cell: (a) $MnCO_3$ (b) $Mn_{0.80}Ni_{0.20}CO_3$ and (c) $Mn_{0.70}Ni_{0.30}CO_3$ and nanospheres electrodes at diverse scanning rates (10 to 100 mV s^{-1}).

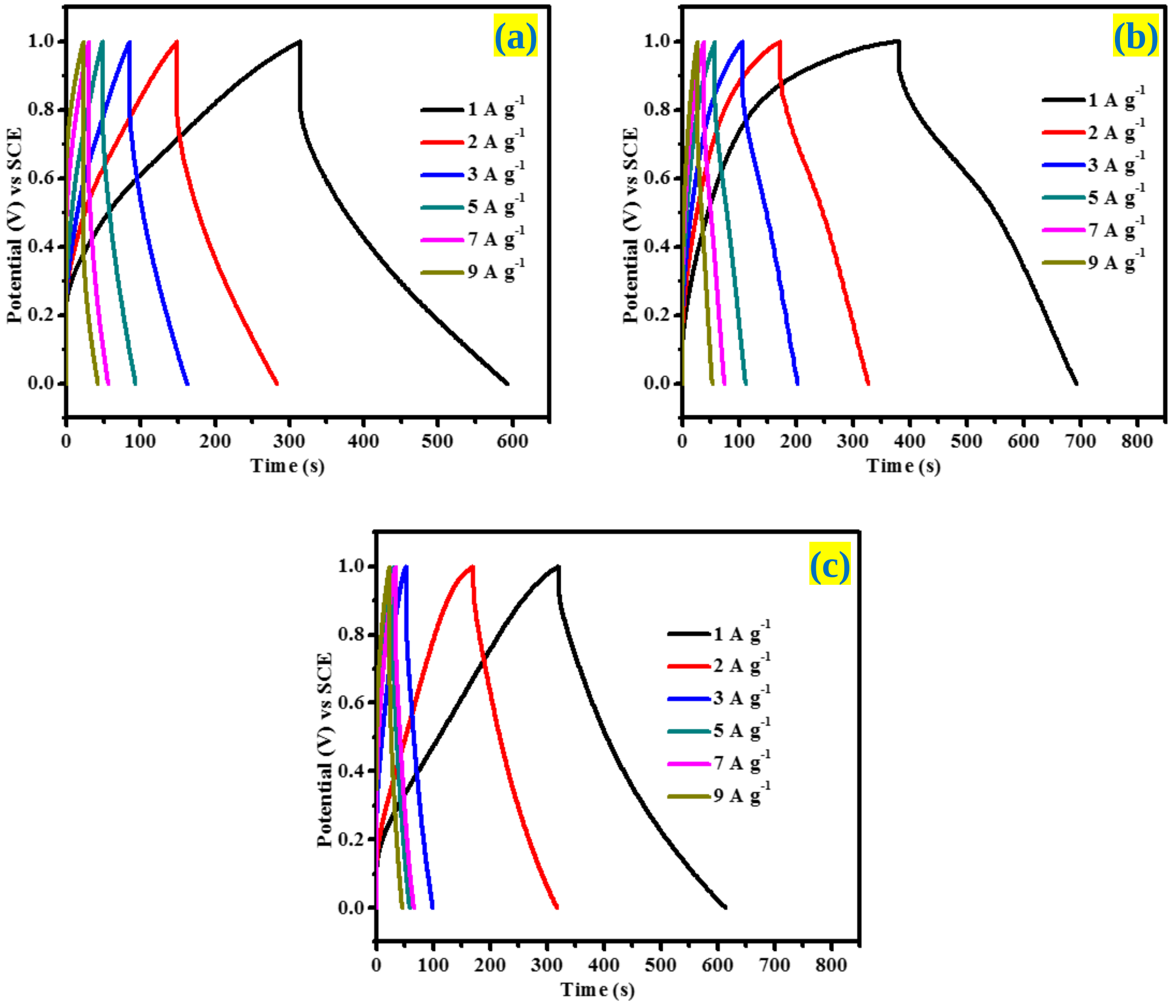

Figure S3. Electrochemical performance of the synthesized samples at 3-electrode cell of GCD curves (a) $MnCO_3$, (b) $Mn_{0.80}Ni_{0.20}CO_3$ and $Mn_{0.70}Ni_{0.30}CO_3$ and nanospheres electrodes at diverse current density (1 to 9 A g^{-1}).

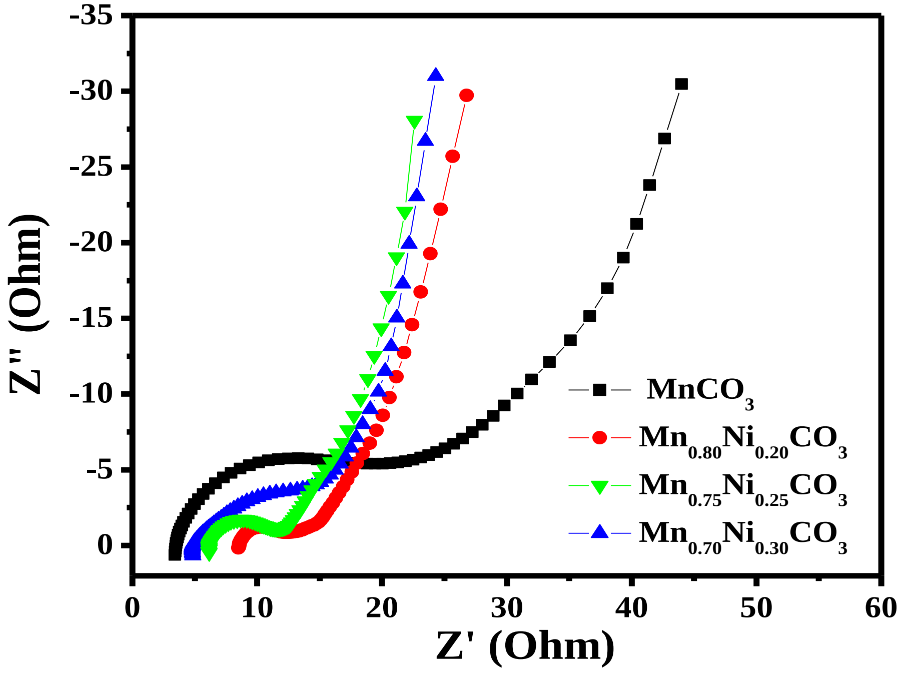

Figure S4. The electrochemical impedance spectra (EIS) of $MnCO_3$, $Mn_{0.80}Ni_{0.20}CO_3$, $Mn_{0.75}Ni_{0.25}CO_3$ and $Mn_{0.70}Ni_{0.30}CO_3$ nanospheres electrodes.

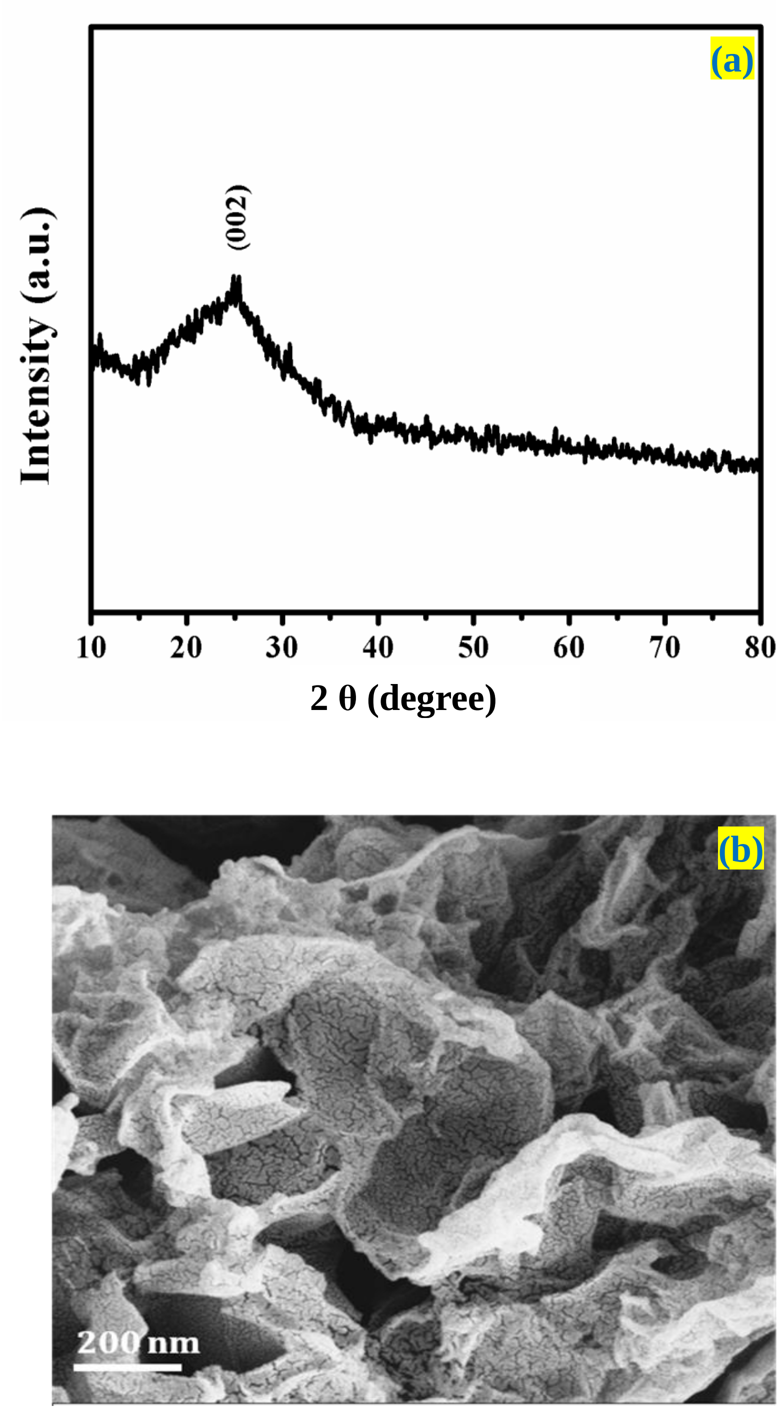

Figure S5 (a) XRD patteren (b) SEM images of GNS

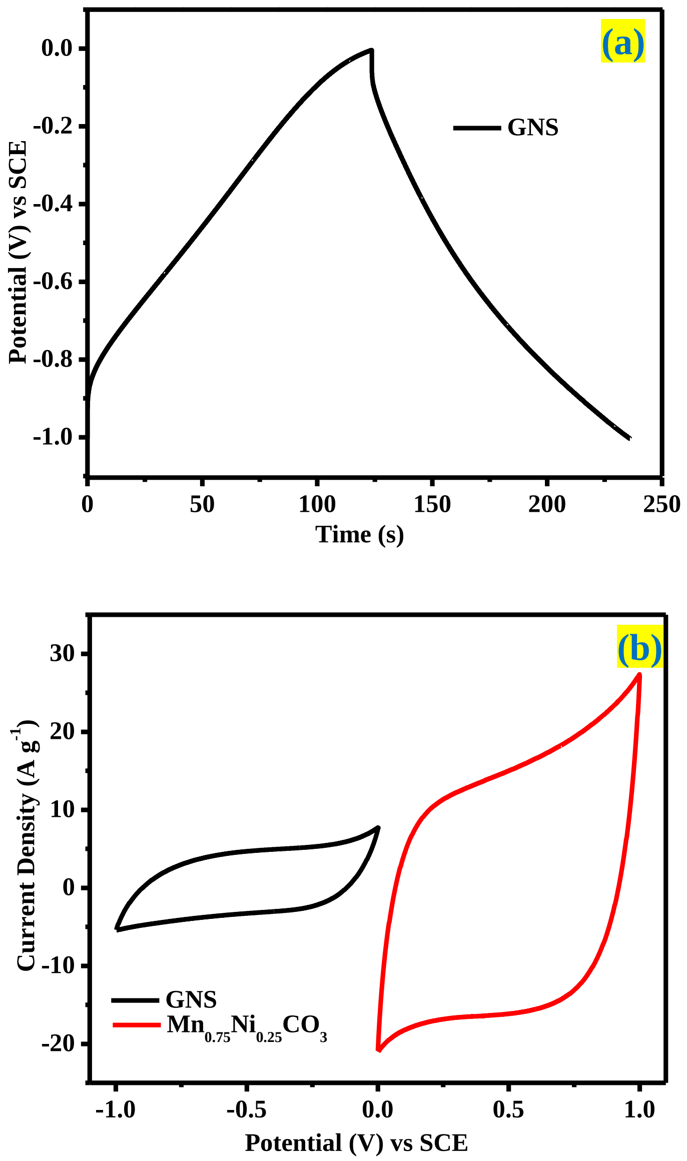

Figure S6 (a) charge discharge curve of GNS at a current density of 1 A g^{-1}, (b) CV curves of GNS (-1.0 V – 0.0 V) and $Mn_{0.75}Ni_{0.25}CO_3$ (0.0 V - 1.0 V) at a scan rate of 10 mV s^{-1}.
